# Phase Evolution by Annealing of Mechanically Activated Ni, Mn, and Sn Elemental Powders Mixture with the Ni_2_MnSn Heusler Compound Ratio

**DOI:** 10.3390/ma18245642

**Published:** 2025-12-15

**Authors:** Florin Popa, Andra Teodora Anastasia Man, Traian Florin Marinca, Ionel Chicinaș

**Affiliations:** 1 Materials Science and Engineering Department, Technical University of Cluj-Napoca, 103-105 Muncii Avenue, 400641 Cluj-Napoca, Romania; teodoraman@yahoo.com (A.T.A.M.); traian.marinca@stm.utcluj.ro (T.F.M.); ionel.chicinas@stm.utcluj.ro (I.C.); 2 EUT+ Institute of Nanomaterials & Nanotechnologies-EUTINN, European University of Technology, European Union

**Keywords:** Heusler alloys, nanocrystalline, mechanical alloying, phase evolution

## Abstract

A Ni_2_MnSn Heusler alloy composition of elemental powders was high-energy milled for a short time for powder activation. The milling times were chosen to be 1 and 4 h to study how mechanical mixing triggers the phase formation in the Ni-Mn-Sn system. After milling, the samples were analyzed by differential scanning calorimetry and the thermal events of Ni_2_MnSn L_21_ phase formation were investigated. The milled samples were compacted at 700 MPa and annealed in a vacuum for 10 min at different temperatures (230 °C, 330 °C, and 600 °C). The annealing temperatures were chosen to emphasize the activated powders’ behavior before and after Sn melting on L_21_ Structure formation. Using X-ray diffraction and Rietveld analysis, the phase quantity was computed, showing that the largest L_21_ phase (63%) can be obtained from the elemental powder mixture due to Sn melting during the annealing. For milled samples, a Ni_3_Sn_4_ phase was obtained by milling, and by annealing this phase, along with the remaining element, it reacts to form a Ni_2_MnSn L_21_ phase and a Ni_3_Sn_2_ phase. The microstructural evolution of the phase was illustrated by backscattering electron microscopy for milled and subsequent annealed samples, and, by image analysis, a correlation of the phase’s amount was performed. The results of the image analysis were correlated with the X-ray diffraction patterns.

## 1. Introduction

Heusler alloys, discovered in the early 20th century, are a class of multifunctional materials with promising applications in fields such as spintronics and thermoelectric and energy technologies [1]. This class of alloy is attracting scientific interest because the properties are given by its structure, since in some cases the alloy has magnetic properties even if its constituent atoms are nonmagnetic [2]. These ternary alloys can exist in two slightly different stoichiometries: 1-1-1, called Half Heusler, or 2-1-1, called Full Heusler type [3,4]. Assigning into one or another class of material is difficult for the Heusler alloys, since they can be metals or semiconductors, but their properties can be predicted based on the valence electrons number [5,6,7]. By adjusting the chemical composition, the band gap width can be adjusted over a wide range (0–4 eV), making them versatile materials with multiple potential uses [2,8,9]. Similarly, the magnetic properties can be tailored by composition or by the valence electron number [7,10,11,12]. In the case of the Heusler alloys, another aspect is found, that the valence electrons number can be influenced by crystallographic transformations and all together modify the transition temperatures [13].

The Heusler alloy’s crystal lattice can be described as face-centered cubic, L_21_ if all atoms are ordered [1]. Having such a complex structure, sometimes it is difficult to obtain the structure and the ways of influencing the properties by applying heat treatments with the purpose of increasing the ordered L_21_ structure are open [1,14]. The ordered structure is the desired one, since in the unordered structures the magnetic properties are weaker [15]. In the large and complex ordered structure, the properties can be tuned by applying pressure on the material, as depicted in reference [16]. Furthermore, atomic order and structure influence the transport properties (electrical resistivity, Seebeck coefficient, thermal conductivity) [17].

Heusler alloys can be obtained by several synthesis methods, the most commonly used being electric arc melting [14] and mechanical alloying [18,19,20,21]. Other methods of obtaining the alloys are rapid quenching [22], thin films [23], or by laser-induced melting [24].

The benefits of Ni_2_MnSn composition are of particular interest, since it was found to possess a high magnetic moment (about 4µ_B_) [25,26], a value that can be tuned by applying pressure or internal stress. Having this large magnetic moment, given by the Mn atoms’ interaction, and having a Curie temperature close to room temperature [23], makes this composition of particular interest for magnetic refrigeration studies [27,28]. To be of further interest, the properties of the Ni-Mn-Sn alloy are largely tunable by composition shifts [29] or by phase purity [30]. It worth adding that the microstructure of the Ni_2_MnSn Heusler alloys has an important effect on reducing the required magnetic field to produce a larger magnetic refrigeration effect [31]. The research on the formation of the Ni-Mn-Sn phase have also found an undesired effect, namely, obtaining additional Ni-Sn or Mn-Sn phases alongside the desired ordered phase (L_21_) [30].

With the problems occurring by the melting of components, especially for Mn, an alternative is sought, one being found to be mechanical alloying. Mechanical alloying (MA) is an out-of-equilibrium method of alloy synthesis in a solid state. In MA experiments, an elemental powder mixture is activated by the energy transfer through collision events between the balls confined into a vial subjected to high speed [32]. This process involves repeated events of fracture and welding for the powder particles, leading to elemental mixing and eventually to the elements’ reaction [33].

The preparation of the Ni_2_MnSn by mechanical alloying was tested and it was found that, in the first hours of milling, a broadening of the elemental powders occurs, followed by a reaction and formation of the Heusler phase [34]. By switching the milling parameters, a more complex formation is found in [35], where, for short milling times, intermediate Ni-Sn phases are reported. It was found that annealing enhances the L_21_ Heusler structure formation, but the undesired Ni-Sn phase cannot be removed [35].

The aim of this research is to fill the gap in the formation study by mechanical alloying, looking at very short-milled samples (0, 1, and 4 h). Another direction is to apply heat treatment near the Sn melting point to observe the effect of liquid Sn on the L_21_ Heusler structure formation for these short milling times. The study thus analyses the effects of powder activation and fresh surface creation by high-energy milling and subsequent annealing. By this approach, we try to understand the kinetics of the Ni-Sn phase formation and to gain a control mechanism that will lead directly to Ni_2_MnSn Heusler phase formation, without additional NixSny phases.

The annealing times are applied for a short duration for the whole sample volume thermal homogeneity only, and not to develop other thermal events. To properly facilitate the reaction of the samples, the powders were compacted before annealing. Since multiple phases were obtained, by milling and temperature-combined effect, phase microstructures are described, visualizing the phases mixing and their modification by low-temperature heating.

## 2. Materials and Methods

Elemental Ni (Vale, 3.5–4.5 µm, 99.8% metal basis), Mn (Alpha Aesar,−325 mesh, 99.3% metal basis) and Sn (Alpha Aesar,−325 mesh, 99.8% metal basis) powders in the Ni_2_MnSn Heusler alloy composition were short milled in a planetary ball mill, Pulveristte 6 Fritch, operating at 350 rpm main disk speed. For the milling experiment, a 250 mL volume hardened steel vial was used, filled with 20 hardened steel balls with 14 mm diameter. The ball-to-powder mass ratio (BPR) was chosen to be 7.2:1 and to avoid oxidation, and the milling was performed in argon atmosphere.

The milled samples were compacted at 700 MPa pressure in cylindrical shapes and subsequently heated at 230 °C, 330 °C, and 600 °C for 10 min in a vacuum. The selected annealing temperatures were chosen based on the differential scanning calorimetry (DSC) curves and this will be explained in the discussions of Figure 2. For the DSC experiments, Labsys Setaram Equipment (Setaram Instrumentation, Caluire-et-Cuire, France) was used. The samples were heated in argon atmosphere flux up to 600 °C with a rate of 10 °C/min. The structural studies were performed by X-ray diffraction, using an INEL 3000 Equinox diffractometer (INEL SAS, Artenay, France), operating with a CoKα wavelength (1.7906 Å) in the 20–110 two theta range. The diffraction patterns were analyzed by means of the Rietveld method [36,37] implemented in the Winplotr software (version April 2023) [38,39].

Scanning electron microscopy (Jeol JSM 5600LV) (JEOL Ltd., Tokyo, Japan) was conducted using a backscattering signal to visualize the microstructures which resulted after annealing. On the recorded images, chemical analysis and elemental map distribution were recorded using an UltimMAX 65 Energy dispersive X-ray spectrometer (EDX) from Oxford Instruments (Oxford Instruments NanoAnalysis, High Wycombe, UK). The data were collected and analyzed by Aztec 4.2 software. The scanning electron microscopy (SEM) images were processed by ImageJ software (version 1.54g) to compute the phase weight for each sample.

## 3. Results and Discussion

**Figure 1:**
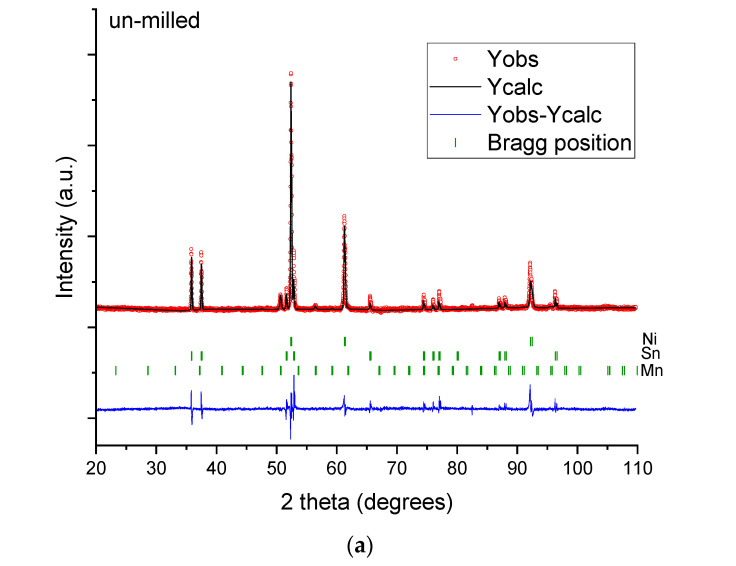

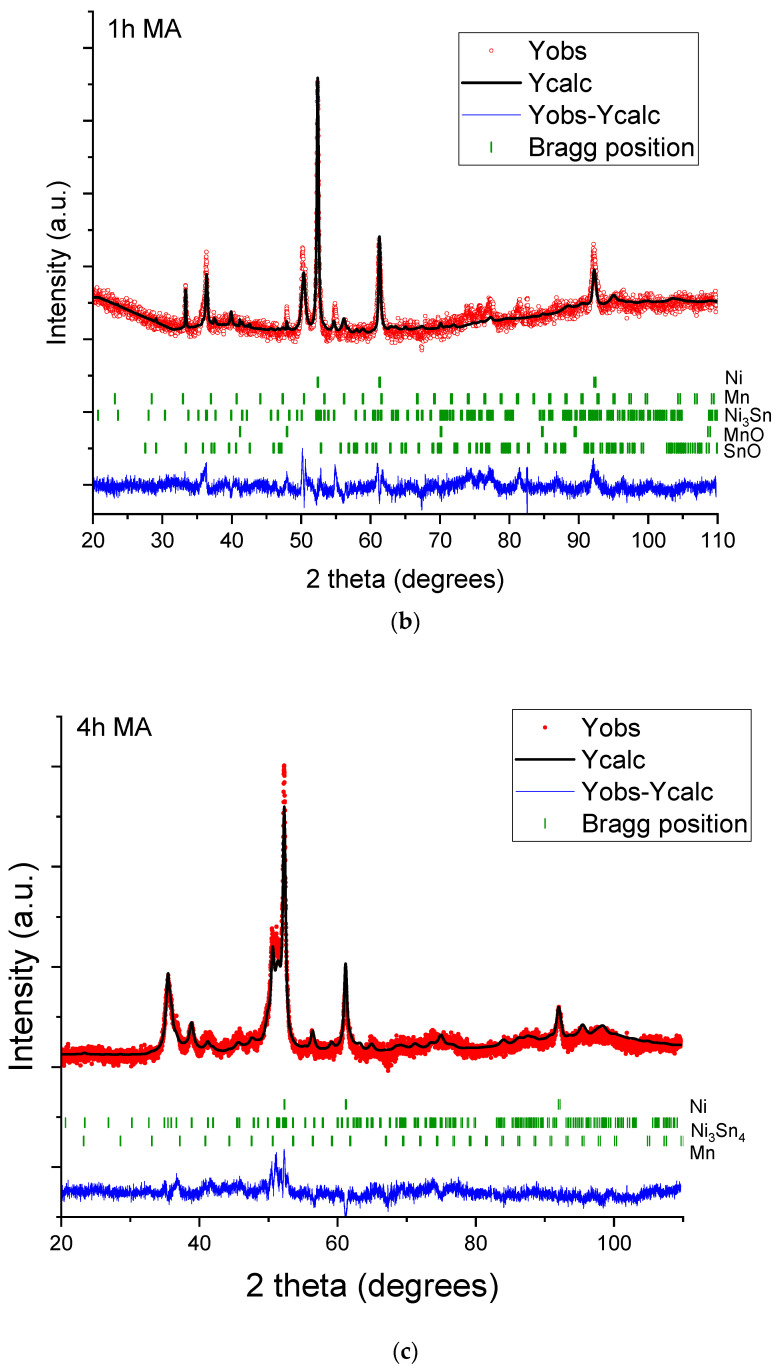
X-ray diffraction patterns for the Ni_2_MnSn Heusler alloy milled (**a**) 0 h, (**b**) 1 h, (**c**) 4 h.

The effect of short milling times on the elemental Ni, Mn, and Sn powders is presented in Figure 1. In this figure, a clear evolution of diffraction peaks is seen from the initial mixture to 4 h of milling.

In Figure 1a, all the diffraction peaks for the elemental powders are seen for the unmilled sample. As the milling is started, the elemental powders start to be mixed and the intensity of the milling leads to the initiation of a solid-state reaction between Ni and Sn, and by this the Ni_3_Sn_4_ phase is obtained. The reaction is initiated by the fracture event on the initial powder particles, which create fresh surfaces with increased reactivity. This observation is confirmed by the affinity of Sn and Ni and its formation energy [40]. Unfortunately, this type of fresh un-passivated surface had a negative impact, since the surfaces of Sn and Mn are prone to oxidize, as the indexation of the diffraction pattern indicates. After 1 h of milling, the diffraction peaks of Sn have disappeared, with only the peaks of Ni and Mn being recorded. The effect of milling from 0 to 4 h has a different impact on the powders. Now, only two phases are recorded, the initial Ni phase and the newly obtained Ni_3_Sn_4_ one. The Mn powders are therefore fragmented and incorporated into the Ni and Ni_3_Sn_4_ matrix.

**Figure 2:**
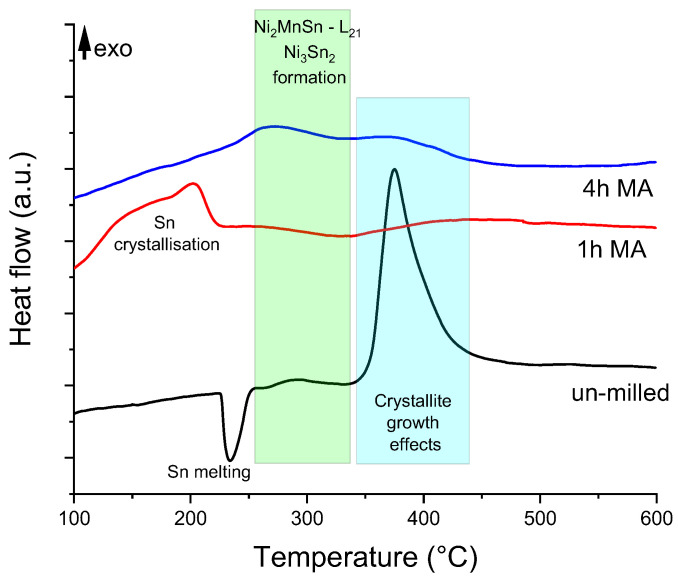
Differential scanning calorimetry curves recorded at heating for the elemental mix of powders, 1 and 4 h milled samples of Ni_2_MnSn Heusler alloy.

Using these three powders, annealing was considered. To properly choose the annealing temperature, DSC curves were recorded. The obtained curves are presented in Figure 2.

The recorded DSC curves show three different behaviors for the samples, depending on their milling period. The un-milled sample is expected to show the Sn melting at a temperature of about 230 °C, as recorded. Further behavior of this sample is controlled by the reaction of melted Sn with Ni and Mn. At the beginning, the reaction forms two compounds: the Ni_2_MnSn Heusler and the Ni_3_Sn_2_, followed by a very sharp exothermic event, at 375 °C, associated with the crystallite growth of these phases. The sample milled for 1 h has a different path; first, the Sn mixed by milling develops crystallite growth, as the exothermic thermal event at 200 °C indicates. This event is then followed by another one, assigned to the Ni_3_Sn_2_ phase formation, a very broad one. The last thermal event is associated with recrystallisation, probably for the phases containing Mn.

The third curve, for the 4 h milled sample, since it is milled for a longer time, has a first peak around 270 °C, corresponding to the Ni_3_Sn_2_ and Ni_2_MnSn phase formation. The curve has a second peak corresponding to the recrystallisation temperature of the above formed phases [35,41].

Based on the DSC measurements, the annealing temperatures were chosen to be at 230 °C (before Sn melts), 330 °C (after Sn melting), and 600 °C (after crystallite growth temperature).

**Figure 3:**
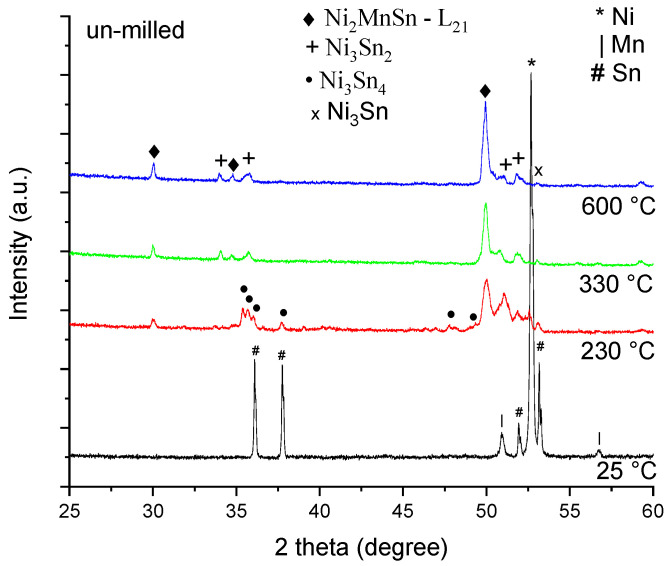
Un-milled sample X-ray diffraction patterns for the Ni_2_MnSn Heusler ratio powder mixture, annealed for 10 min in vacuum at 230 °C, 330 °C, and 600 °C.

Figure 3 shows the phase evolution of the un-milled sample, annealed for 10 min at different temperatures.

Analyzing the diffraction patterns by means of the Rietveld refinement, the following phases and amounts were found, as presented in Table 1.

Table 1 shows the elemental powder reaction as the temperature increases, with a sharp change as the Sn is melting at around 235 °C. As the Sn melts, it creates conditions to promote a reaction between the elements, and the Ni_2_MnSn Heusler compound is formed. As a secondary phase, the Ni_3_Sn_4_ one is also formed [35,41]. Heating above Sn melting temperature, a larger amount of the Ni_2_MnSn phase is formed, reaching more than 55% of the sample, and the Ni_3_Sn_2_ phase is promoted as a secondary phase, instead of Ni_3_Sn_4_. Heating up to 600 °C has the effect of forming more of the Ni_2_MnSn phase (up to 63%) and consuming the elemental powders. At this heating temperature, only the Mn and Ni phases are visible.

**Figure 4:**
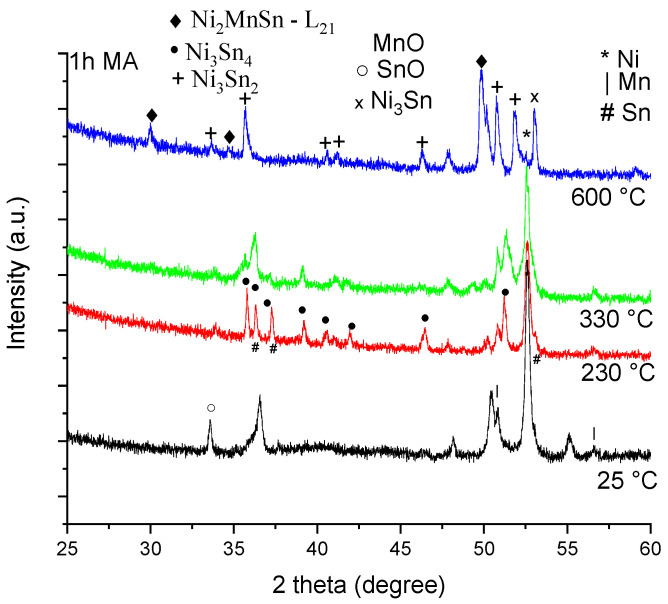
X-ray diffraction patterns for the Ni_2_MnSn sample milled for 1 h, and annealed in vacuum for 10 min at 230 °C, 330 °C, and 600 °C.

Figure 4 shows the phase recorded upon annealing the 1 h milled sample, with the purpose of investigating the effect of mechanically activated powders.

Analyzing the diffraction patterns by means of the Rietveld refinement, the following phases and amounts were found, as presented in Table 2.

The phases resulting from 1 h of milling and subsequent annealing are very interesting, reflecting the initial stage of mechanical alloying, namely, the powder fracture and the exposing of a fresh surface ready to react. Therefore, the 1 h milled sample is very hard to handle in air, since the fresh surfaces of Sn and Mn will quickly react with air to form oxides [42]. However, a careful handling in a protective atmosphere, followed by annealing, overcomes this sensitivity of the Mn in particular. By careful handling, after annealing at 230 °C, the unreacted Sn melts and is again visible in the diffraction patterns, and now the only promoted phase is Ni_3_Sn_4_. After annealing at 330 °C, the Ni_3_Sn_2_ phase also starts to form, as does the Ni_2_MnSn phase, in a very small amount. Increasing the temperature up to 600 °C, the Ni_2_MnSn phase amount grows, reaching an amount of 36%, alongside the Ni_3_Sn_2_ phase (36%) and the Ni_3_Sn phase (17%). At this temperature, a larger MnO phase occurs. At first, it seems that activating the powders increases the energy of the Ni_2_MnSn phase formation, since an amount of thermal energy is directed to the initial powder recrystallisation (Sn) and the internal stress removal induced by milling.

**Figure 5:**
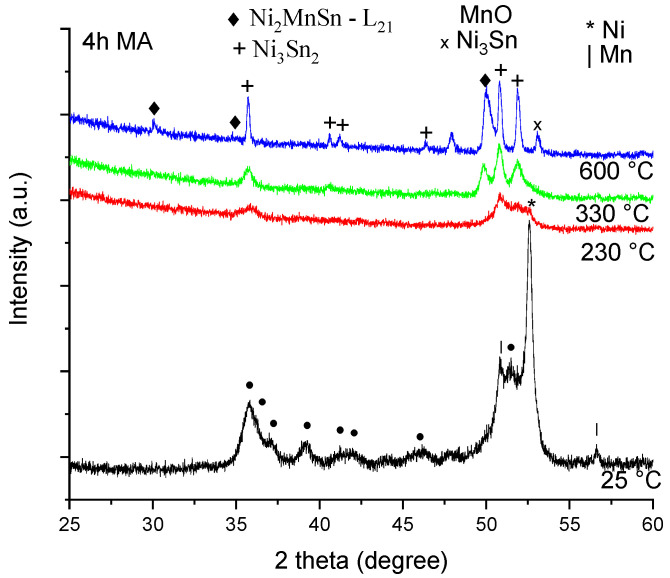
X-ray diffraction patterns for the Ni_2_MnSn sample milled 4 h, and annealed in vacuum for 10 min at 230 °C, 330 °C, and 600 °C.

The phase evolution of the 4 h milled and subsequently annealed sample is presented in Figure 5.

Analyzing the diffraction patterns by means of the Rietveld refinement, the following phases and amounts were found, as presented in Table 3.

Milling the sample up to 4 h leads to complete Sn disappearance and the formation of the Ni_3_Sn_4_ phase. Heating the sample at 230 °C leads to a complete Mn reaction and the formation of a large Ni_3_Sn_4_ quantity (about 72%). There is visibly a large shift in the phases between the as-milled and 230 °C annealed sample, where the consumption of the Mn and Ni is clearly visible. Continuing the heating at 330 °C, all elemental powders are reacted. The Ni_2_MnSn phase germinates as well, and the Ni_3_Sn_4_ phase is transformed into the Ni_3_Sn_2_ phase. At an even higher temperature, the formation of the Ni_2_MnSn phase continues, reaching 37% by consuming the Ni_3_Sn_2_ phase. Unfortunately, other secondary phases are obtained (Ni_3_Sn and MnO).

Analyzing the data obtained after DSC and X-ray diffraction studies upon the annealed samples, a reaction kinetic can be deduced (Equation (1)). The data from our study are correlated with other available data on the compounds’ formation energy in the Ni-Sn system [19,43,44].

Ni_3_Sn_4_ + Mn + Ni → Ni_3_Sn_2_ + Ni_2_MnSn (1)

The findings are consistent with the behavior of the powders in the first hours of milling, where a small Sn layer is formed on the Ni particles, leading to Ni_3_Sn_4_ formation, as reported in [45], followed by increasing the contact surface between Ni_3_Sn_4_ and Ni particles. In this stage, the low temperature can initiate the Ni_3_Sn_2_ phase formation [46]. At the same time, by adding Mn, the Heusler phase Ni_2_MnSn is obtained.

In conclusion, the thermal treatment of the samples has a large effect on the unmilled sample, leading to a large Ni_2_MnSn Heusler compound. By milling, the activation formation of the Ni_2_MnSn Heusler phase is hindered, since lower quantities are obtained at similar temperatures. But the milling has a positive effect on reducing the Ni-Sn phase number present in the milled and annealed samples.

**Figure 6:**
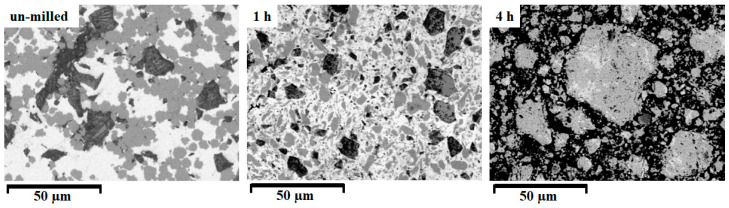
Backscattered scanning electron images for un-milled, 1 and 4 h milled Ni_2_MnSn Heusler alloy samples, ×1000.

The mixing and the reaction of the elemental powder can be observed easily by scanning electron microscopy, using a backscattered signal for the milled samples, as presented in Figure 6.

The electron images, exposing the compositional contrast, show for the un-milled sample a compact and sharp color for each element: with gray for Sn, dark gray for Ni, and black for Mn. Due to the components’ plasticity, no pores were observed for the un-milled and 1 h milled samples. As the milling starts, the distribution of colors changes, since the elemental powders are mixed and incipient reactions occur between them. The particles at this stage are rather large, since the cold-welding events predominate. After 4 h of milling, a different picture is seen: the whiter areas have almost disappeared, attesting to the Sn reaction, and a new gray area is observed, corresponding (as the X-ray diffraction indicates) to a Ni_3_Sn_4_ alloy. Some small areas with gray (corresponding to unreacted Ni) and black (corresponding to unreacted Mn) are still visible. It is worth noting that after 4 h of milling the particle size is reduced, compared with the 1 h milled sample.

Next, the milled samples were analyzed by scanning electron microscopy for different annealing temperatures.

**Figure 7:**
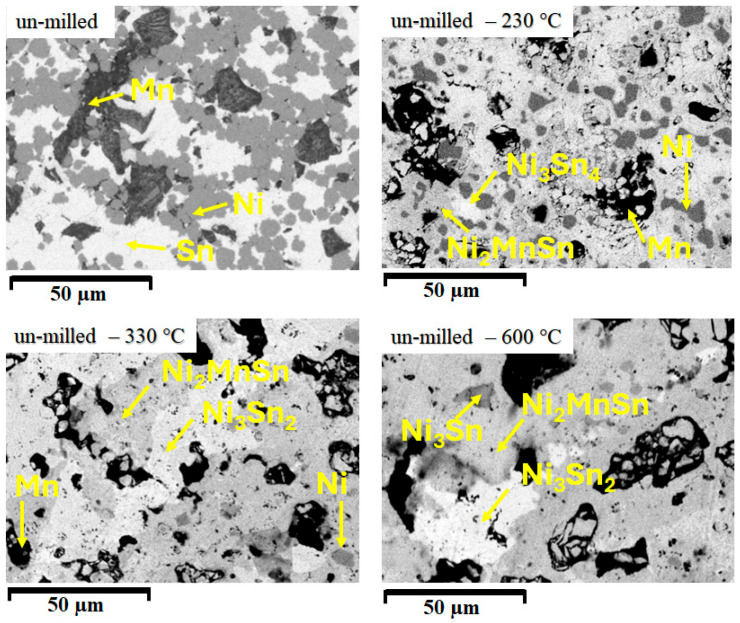
Scanning electron microstructures for the un-milled Ni_2_MnSn Heusler alloy and for compacted sample, annealed at 230 °C, 330 °C, and 600 °C.

Figure 7 shows the microstructures obtained for the un-milled sample.

The phase distribution confirms the results of the X-ray diffraction study and shows how a reaction occurs upon heating the initial mixture of powders. After 10 min of annealing at 230 °C, a temperature very close to the pure Sn melting temperature has as an effect the reaction of Ni with Sn and the formation of a Ni-Sn alloy. Near the Sn melting temperature, the heating energy provides sufficient energy to promote a reaction between the elements, leading to the formation of the Ni_2_MnSn and Ni_3_Sn_4_ phase. Upon heating above the Sn melting temperature, the Ni_3_Sn_4_ phase transforms into the Ni_3_Sn_2_ phase as an effect of formation energy change [40], and, by consuming the Mn areas, extends the area size of the Ni_2_MnSn Heusler phase. When the temperature increases to 600 °C, the Heusler phase becomes the dominant phase, surrounding areas of Ni_3_Sn_2_ and Ni_3_Sn phase that are also forming at his temperature. As a short conclusion, only by heating at 600 °C a compacted mixture of Ni, Sn and Mn powders can form a large amount of Ni2MnSn Heusler phase.

**Figure 8:**
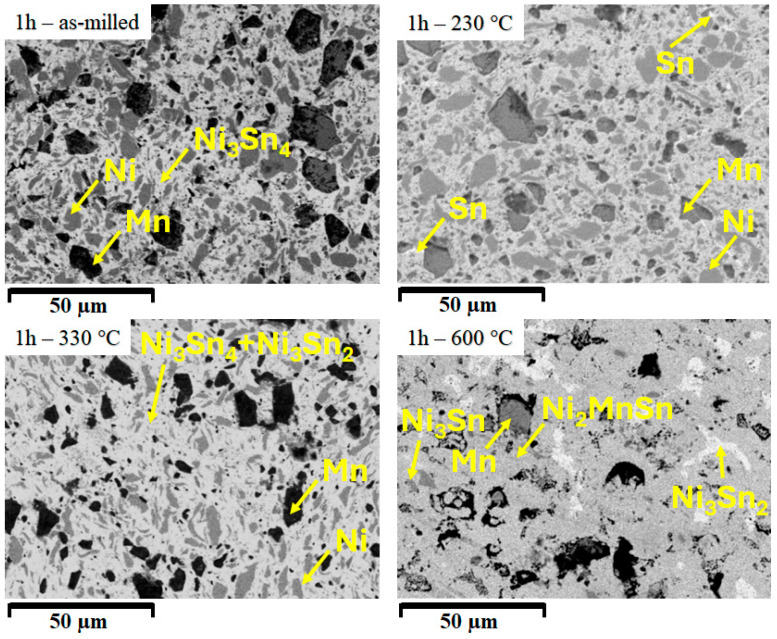
Scanning electron microstructures for the 1 h Ni_2_MnSn Heusler alloy milled and compacted sample, annealed at 230 °C, 330 °C, and 600 °C.

The picture of the thermal influence on the powders is now studied (Figure 8) by applying the heat treatment to a mechanically activated sample, that is to say, the sample milled for only 1 h.

At 1 h of milling, the as-milled sample shows a mixture of Ni, Mn, and Ni_3_Sn_4_. The larger part of the sample is composed of the Ni_3_Sn_4_ phase, which embeds clusters of Ni and Mn. Heating the sample at 230 °C has the effect of increasing the Ni_3_Sn_4_ quantity, but also Sn recrystallisation, as indicated by some faint white areas in Figure 8. Increasing the temperature up to 330 °C modifies the phase content, but at a microstructural level only the Sn areas have disappeared. At 600 °C, the temperature forms the Heusler phase, Ni_2_MnSn by dissolving the remaining Ni and has the effect of clustering Ni_3_Sn_2_ phase and the formation of Ni_3_Sn phase.

**Figure 9:**
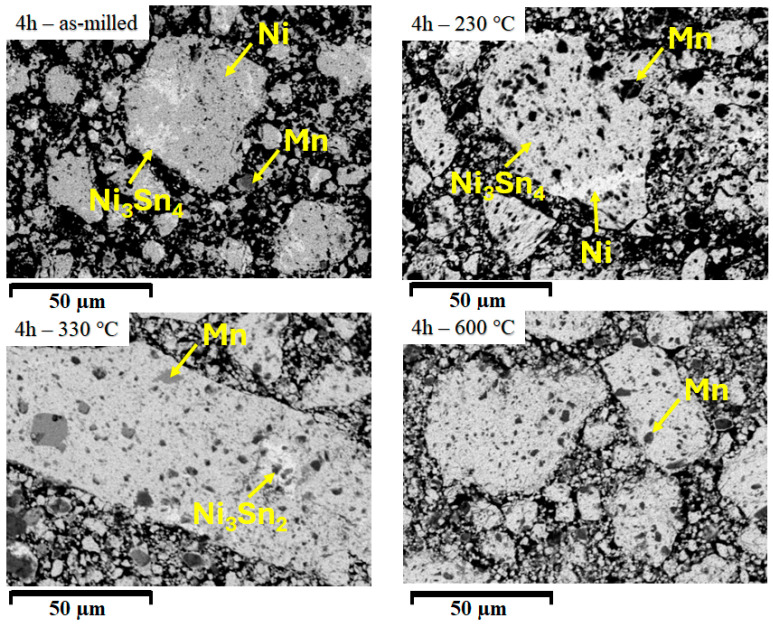
Scanning electron microstructures for the 4 h Ni_2_MnSn Heusler alloy milled and compacted sample, annealed at 230 °C, 330 °C, and 600 °C.

The formation of the phases for the 4 h milling time sample is less dynamic than in previously milled samples, as the images from Figure 9 display.

For the as-milled sample, three types of areas are visible, corresponding to elemental Ni, Mn, and the Ni_3_Sn_4_ formed phase. Heating at 230 °C and 330 °C has little impact on the observed phases, except that, as the X-ray diffraction has shown, the Ni_3_Sn_4_ phase is replaced by the Ni_3_Sn_2_ one. All the samples have Mn clusters, which do not react by the combined action of milling and temperature.

**Figure 10:**
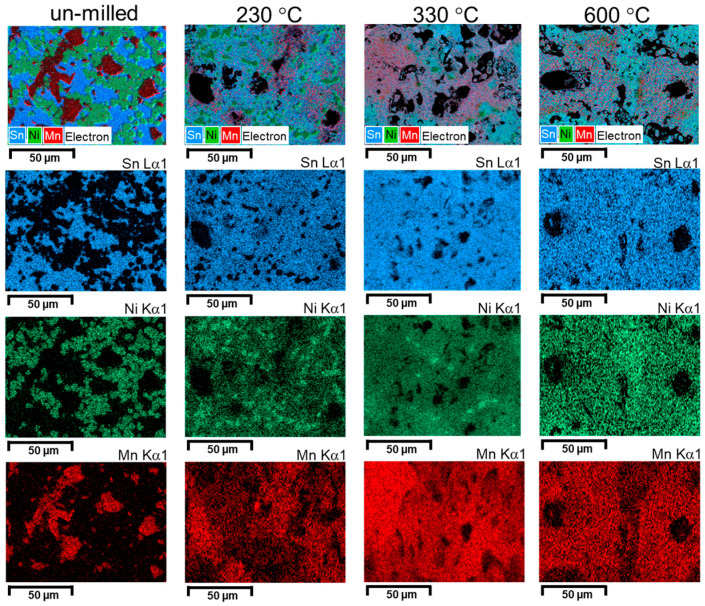
Elemental map distribution for Ni, Mn, and Sn for the Ni_2_MnSn un-milled sample and annealed at 230 °C, 330 °C, and 600 °C.

**Figure 11:**
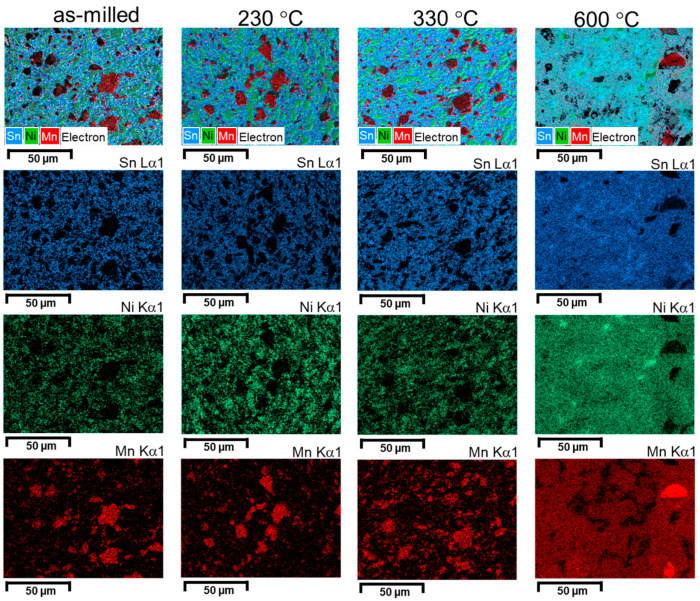
Elemental map distribution for Ni, Mn, and Sn for the Ni_2_MnSn sample milled 1h and annealed at 230 °C, 330 °C, and 600 °C.

**Figure 12:**
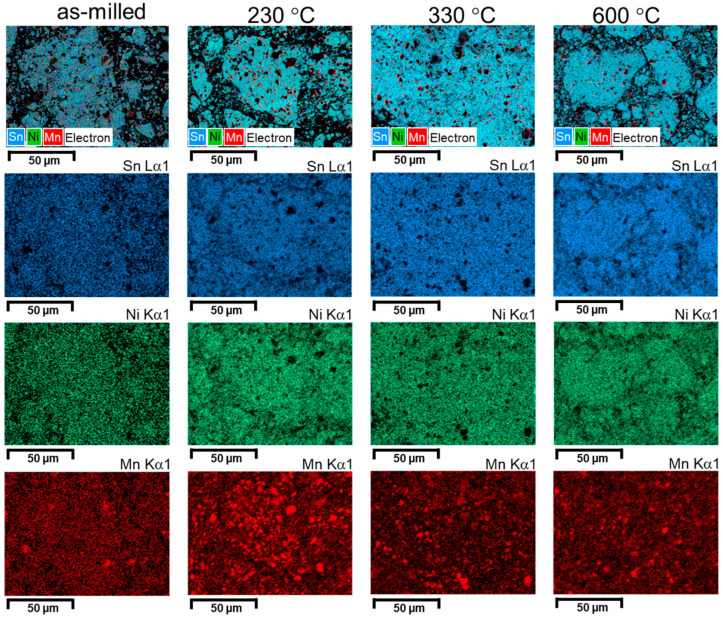
Elemental map distribution for Ni, Mn, and Sn for the Ni_2_MnSn sample milled 4 h and annealed at 230 °C, 330 °C, and 600 °C.

The evolution of the phases can be visually described by showing elemental map distribution images (Figure 10, Figure 11 and Figure 12).

Analyzing the elemental distribution maps, it can be seen that, for the un-milled sample, the annealing has the effect of consuming the Ni particles. This effect is visible by the size reduction in the Ni areas. In addition, the diffusion is seen by the Mn area extension on the analyzed area. However, at high temperature (600 °C), a new agglomeration of Ni particles can be seen, revealing Ni_2_MnSn phase formation. As the milling is started, and a good dispersion of elemental powders particles is made, increasing the temperature has the effects of phase formation between Ni and Sn, and Mn clustering. If the milling is even higher (4 h), the dispersion by milling is enough to homogenize distributions for Sn and Ni and to reduce the size of the Mn clusters. As a short conclusion, the study of elemental distribution maps as a function of milling and annealing visualizes the effect of completing the elemental reaction, especially when elemental Sn exists in the sample if the temperature is higher than its melting temperature. For the milled sample, a clustering effect is noticed when passing from 330 °C to 600 °C. The effect is more visible for Mn.

**Figure 13:**
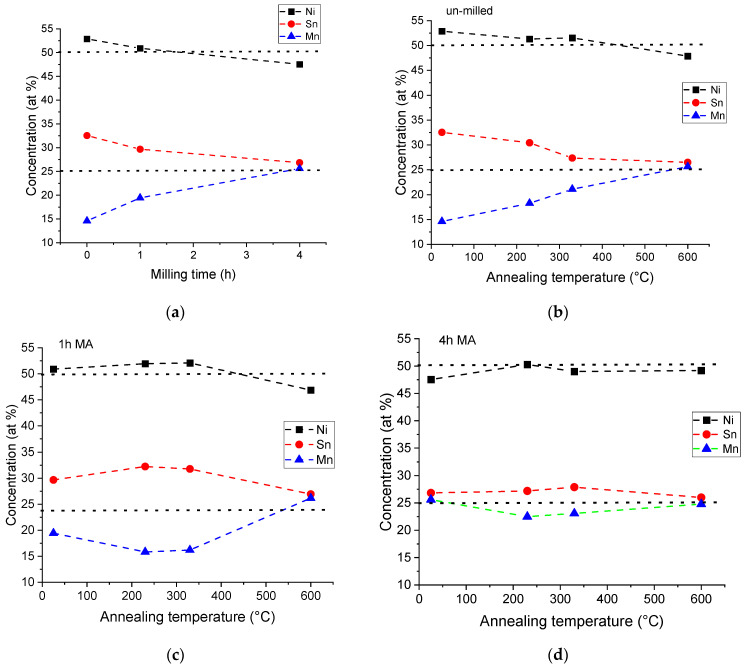
Local area chemical analysis of the compacts for different milling times (**a**) and for different temperatures of the annealed sample, un-milled sample (**b**) and milled for, (**c**) 1 h and 4 (**d**) 4 h, respectively.

Since several local chemical analyses were performed to properly assign the observed phases in the microstructural images, a plot of the global amounts of the percent of each element was computed. The evolution of elemental quantity distribution with milling time and annealing temperature was computed, as Figure 13 shows.

From this analysis, it is visible that, as the milling time is increasing, the composition goes towards the desired one. Concerning the annealing temperature and milling time, the process is similar; only the way the composition reaches the desired one is slightly different.

**Figure 14:**
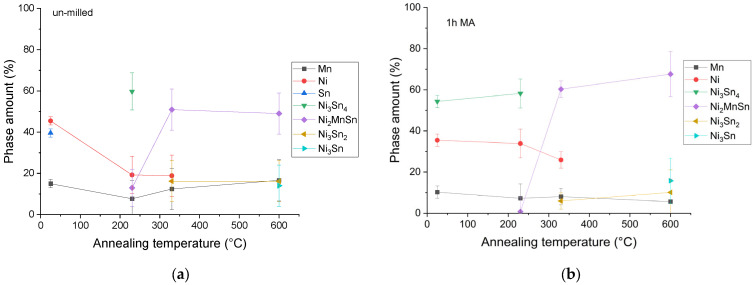
Phase amount evolution as computed by phase color distribution for the un-milled (**a**) and 1 h milled sample (**b**) and annealed at different temperatures.

For the un-milled and 1 h of milling, the phases are plotted versus temperature in Figure 14. Since the 4 h milled sample has smaller variation in compositions and contains a large porosity (up to 20% of the sample), its phase ratio was not computed by this method.

The computed phases underline the continuous decrease in the elemental powder ratio and the increase in the Ni-Sn phase, and the Ni_2_MnSn Heusler one in particular. For samples annealed at 600 °C, the Heusler phase reaches values higher than 50–60% of the sample.

## 4. Conclusions

Ni_2_MnSn Heusler alloy formation was studied by using a combined method of short mechanical alloying and subsequent low-temperature annealing. By short milling, powder particle surface activation was promoted, leading to elemental mixing and reaction. The thermal study of the milled samples showed that, in the very short milling times, a Sn crystallization occurs and the formation of the Ni_2_MnSn L_21_ structure is an exothermic event, occurring around 300 °C. The melting of Sn in the un-milled sample has a large impact on the formation of the Ni_2_MnSn L_21_ phase; this phase can reach up to 63% if the annealing is performed at 600 °C for 10 min. In the milled samples, first, the Ni_3_Sn_4_ phase is formed, and, by annealing, this phase and the unreacted elements transform into the Ni_3_Sn_2_ and Ni_2_MnSn L_21_ phase. The microstructural evolution of the phase was illustrated by backscattering electron microscopy for milled and subsequent annealed samples, and, by image analysis, a correlation of the phases was performed. The results of the image analysis are well correlated with the X-ray diffraction patterns.

## Figures and Tables

**Table 1:** The identified phases and computed amounts for the un-milled Ni_2_MnSn sample.

Annealing Temperature (°C)	Ni (wt. %)	Sn (wt. %)	Mn (wt. %)	Ni_2_MnSn L_21_ (wt. %)	Ni_3_Sn_4_ (wt. %)	Ni_3_Sn_2_ (wt. %)	Ni_3_Sn (wt. %)
**as mixed**	40.3	40.8	18.9				
**230**	10.9	1.7	27.6	30.0	29.8		
**330**	5.8		14.2	55.6	2.8	18.4	3.2
**600**	8.3		7.4	63.3	2.9	16.6	1.5

**Table 2:** The identified phases and computed amounts for the 1h milled Ni_2_MnSn sample.

Annealing Temperature (°C)	Ni (wt. %)	Sn (wt. %)	Mn (wt. %)	Ni_2_MnSn L_21_ (wt. %)	Ni_3_Sn_4_ (wt. %)	Ni_3_Sn_2_ (wt. %)	Ni_3_Sn (wt. %)	MnO (wt. %)	SnO (wt. %)
**as milled**	53.2		27.4		15.3			1.7	2.7
**230**	48.0	3.4	27.7		21.0				
**330**	32.3		15.1	0.9	24.2	22.0			
**600**				35.9		36.4	17.4	10.3	

**Table 3:** The identified phases and computed amounts for the 1 h milled Ni_2_MnSn sample.

Annealing Temperature (°C)	Ni (wt. %)	Mn (wt. %)	Ni_2_MnSn L21 (wt. %)	Ni_3_Sn_4_ (wt. %)	Ni_3_Sn_2_ (wt. %)	Ni_3_Sn (wt. %)	MnO (wt. %)
**as milled**	43.4	22.9		33.7			
**230**	27.9			72.1			
**330**			23.2		76.8		
**600**			37.7		39.7	8.1	14.4

## Data Availability

The original contributions presented in this study are included in the article. Further inquiries can be directed to the corresponding author.
